# Measuring sensitivity to eye gaze cues in naturalistic scenes: Presenting the eye gaze FoCuS database

**DOI:** 10.1002/mpr.1833

**Published:** 2020-07-14

**Authors:** Gordon Bill, Elisabeth Whyte, Jason W. Griffin, K. Suzanne Scherf

**Affiliations:** ^1^ Pennsylvania State University State College Pennsylvania USA

**Keywords:** autism, face, gaze following, gaze perception, social anxiety

## Abstract

**Objectives:**

The ability to process information about eye gaze and its use for nonverbal communication is foundational to human social interactions. We developed and validated a database of stimuli that are optimized to investigate the perception and referential understanding of shifts in eye gaze.

**Methods:**

The 245 *Gaze Perception* stimuli are digital photographs that test the ability to estimate and interpret eye gaze trajectory. The 82 *Gaze Following* stimuli are digital videos that measure the ability to follow and interpret eye gaze shifts online. Both stimuli were designed for a 4‐alternative forced choice paradigm (4AFC) in which the participant identifies the gazed‐at object.

**Results:**

Each stimulus was validated by independent raters and only included if the endorsement of the correct item was ≥75%. Finally, we provided timestamps for 19 40‐second video segments from adolescent‐oriented entertainment movies that are matched on several factors. These segments involve social interactions with eye gaze shifts and can be used to measure visual social attention.

**Conclusions:**

This database will be an excellent resource for researchers interested in studying the developmental, behavioral, and/or neural mechanisms supporting the perception and interpretation of eye gaze cues.

## 1 INTRODUCTION

Visuoperceptual sensitivity to eyes and the rudimentary ability to follow shifts in eye gaze are present within days of birth in humans (Farroni, Csibra, Simion, & Johnson, 2002; Farroni, Massaccesi, Pividori, & Johnson, 2004). These early proclivities reflect the importance of developing an understanding that “the eyes capture information about the world” (Emery, 2000). As humans, we use shifts of gaze to communicate to each other about the relative importance of objects and people in the world. Interpreting these signals requires referential understanding of visual behavior, which reflects a realization that visual behavior is directed toward objects/content (i.e., it is not abstract in nature) and that it involves the mental experience of seeing something (Moore, 1999; Moore & Dunham, 1995). In other words, it requires establishing a psychological connection between the looker and the content (Brooks & Meltzoff, 2005). During the first 2 years of life, human infants develop increasing referential understanding of eye gaze cues, as they learn that open eyes, not closed eyes or simple head direction, provide communicative information about content (e.g., Brooks & Meltzoff, 2005; Butler, Caron, & Brooks, 2000).

Gaze information is also used to communicate about many aspects of social cognition including deception, empathy, and theory of mind (for review see Emery, 2000). As a result, sensitivity to gaze information allows us to make attributions about the intentions and motivations driving another's behavior, respond to those behaviors, and anticipate subsequent behavior. Therefore, the ability to accurately process information from eye gaze and understand that and how it is used for social communication is foundational to human social interactions.

Given this essential role of eye gaze cues in social communication, there are important questions to address about the emerging sensitivity to these cues over the course of development and understanding the consequences for individuals who have difficulties perceiving and/or interpreting these cues. For example, autism spectrum disorder (ASD) is a developmental disability that impacts social communication and the processing of eye gaze cues (American Psychiatric Association, 2013). Individuals with ASD exhibit atypical eye contact specifically in social interactions, which often manifests as reduced eye contact. Similarly, individuals with social anxiety disorder avoid eye contact (Schulze, Renneberg, & Lobmaier, 2013). For both disorders, there are clear predictions that reduced visual attention to eyes likely compromises the understanding of nonverbal communication cues, like shifts in eye gaze. However, the vast majority of existing research with these populations measures visual attention (i.e., using eye tracking) during passive viewing paradigms without including measures of referential understanding (i.e., communicative intent) of the cues. This is problematic because gaze following could reflect sensitivity to a predictive spatial cue (i.e., head or gaze direction indicates something interesting is about to happen over there, Butterworth & Cochran, 1980) in the absence of comprehension about the psychological relation between the looker and target. Therefore, researchers studying gaze perception and following behaviors must employ strategies to assess referential understanding of gaze cues (i.e., understanding the content of the looker's gaze) in order to assess the integrity of these behaviors.

To facilitate more research on the referential understanding of eye gaze cues, we developed and validated a database of stimuli that include a set of images and videos in which actors are embedded in complex scenes, directing gaze to one of many possible objects. Each item is designed to be tested in a 4‐alternative forced choice (4AFC) task in which participants identify the target gazed‐at object from a list of four labels. In so doing, the referential understanding of the gaze cues is quantified by participants' accuracy. Specifically, in order to perform well in this task and successfully identify the target object, participants must be able to follow gaze *and* understand that the actor is referencing an object with their line of sight. In this article, we review the limitations in the current literature that lead to the development of these stimuli, identify the primary goals and strategies motivating the development of this database, and overview the validation procedure and results.

## 2 LIMITATIONS IN CURRENT RESEARCH

There are several limitations in the current literature investigating perception of eye gaze among both typically developing and atypical populations. These include the lack of publicly available stimuli that are optimized to investigate the perception and referential understanding of eye gaze cues, the reliance on passive viewing paradigms and respective stimuli, and as a result, the lack of stimuli and experimental paradigms that evaluate referential understanding of gaze cues. We will briefly explain these limitations and then describe how the stimuli in the Eye Gaze Following and Cuing Stimuli (FoCuS) database begin to address these gaps in the current literature.

There are databases of stimuli available for researchers investigating multiple aspects of face processing, including identity recognition (Gao, Cao, Shan, Chen, & Zhou, 2008; O'Toole et al., 2005; Phillips, Wechsler, Huang, & Rauss, 1998; Ricanek & Tesafaye, 2006), emotional expression perception (Dalrymple, Gomez, & Duchaine, 2013; Ma, Correll, & Wittenbrink, 2015; Tottenham, Tanaka, Leon, McCarry, & Nurse, 2009) and trait perception (Goeleven, De Raedt, Leyman, & Verschuere, 2008). There is one database that has faces with both direct and averted gaze (Langner, Dotsch, Bijlstra, Wigboldus, & Hawk, 2010); however, in the averted gaze images the actors are not directing gaze to anything in particular, which prevents any investigation of referential understanding of gaze cues. GazeFollow is another publicly available database that is comprised of a collection of natural images culled from existing image databases and rated to determine where the people in the images are looking (Recasens, Kholsa, Vondrick, & Torralba, 2015). Importantly, these images were not specifically designed to measure sensitivity to eye gaze cues. As a result, there is no ground truth information about where gaze is directed and no systematic manipulation of the objects in a scene. Therefore, there appear to be no publicly available databases with stimuli that are designed to test perception and referential understanding of eye gaze cues.

Another limitation in the existing literature is related to the most common approach to studying the perception of eye gaze cues. This involves using eye tracking technology to measure fixation duration and/or scan path trajectory as participants visually observe stimuli (for review see Guillon, Hadjikhani, Baduel, & Rogé, 2014). These stimuli usually include static images (e.g., Freeth, Chapman, Ropar, & Mitchell, 2010) or dynamic videos (e.g., Klin, Jones, Schultz, Volkmar, & Cohen, 2002) of gaze cues and the paradigms typically only require passive viewing from participants. Furthermore, these studies do not include condition manipulations or other measures that evaluate referential understanding of gaze. As a result, researchers using these paradigms measure visual attention to the gaze cues, but not referential understanding of the cues.

Finally, in the developmental disabilities literature, there are a small number of studies that do investigate both visual attention to and referential understanding of eye gaze cues in individuals with ASD and Williams syndrome (Riby & Hancock, 2009; Riby, Hancock, Jones, & Hanley, 2013). In these studies, participants looked at a static image of a person in a complex scene who is directing gaze toward one of many possible objects. Participants were required to generate their own verbal label to describe the target gazed‐at object to indicate referential understanding of gaze. The researchers recorded visual attention via eye tracking as well as the accuracy of the ability to verbally identify the target object. This paradigm inspired us to develop a version of this task in which participants did not have to generate their own verbal response, but could select a response in a 4AFC. In so doing, we could establish chance level performance and minimize the potential influence of differences in verbal skills on task performance.

## 3 THE EYE GAZE FOCUS DATABASE

**FIGURE 1:**
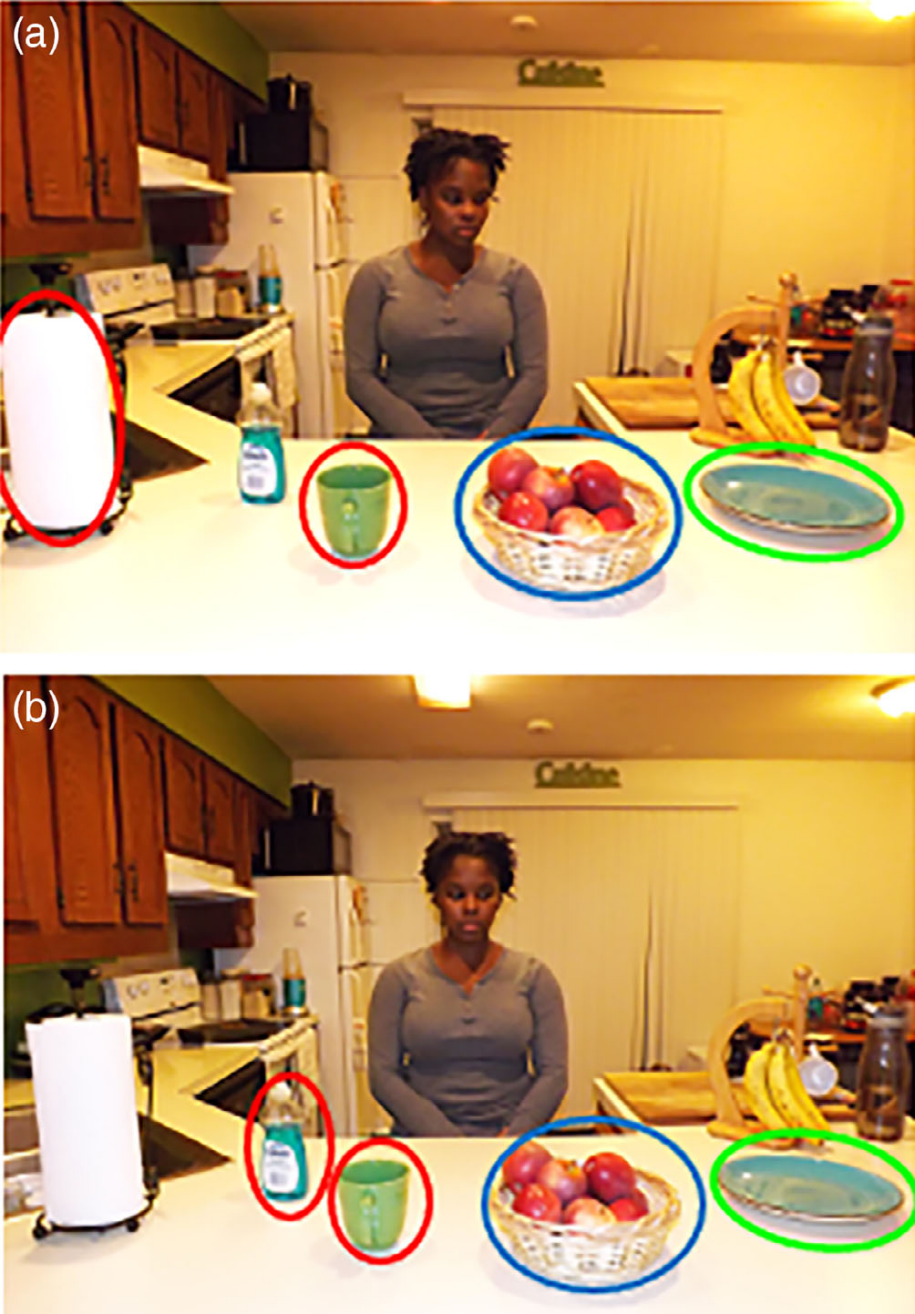
Gaze perception stimuli. Examples of Gaze Perception stimuli. The actor is directing gaze toward one of many possible objects in a complex, naturalistic scene. In half the images, the head and gaze align in the direction of the target object (a). In the other half of the images, the head and gaze cues were misaligned by having the actor keep the head straight forward and only moving the eyes to the target object (b). In both images the target object is circled in green, the plausible nontarget object is circled in blue, and the implausible nontarget objects are circled in red. These two images also represent the notion that the implausible objects can vary across similar scenes

Here, we introduce a database of eye gaze stimuli that we developed to address some of these limitations. The database includes three kinds of stimuli, digital images and videos of actors portraying targeted eye gaze shifts toward objects, and timestamps of segments in commercial entertainment movies that highlight social interactions between adolescents and adults with eye gaze shifts. The Gaze Perception stimuli are designed to test the ability to interpret eye gaze trajectory from photographs like those used in prior studies (e.g., Riby et al., 2013). They include 245 digital color photographs of actors in complex naturalistic visual scenes (see Figure 1a,b). The actor is directing gaze to one of many possible objects. The Gaze Following stimuli are designed to test the ability to follow and interpret shifts in gaze online. The stimuli were modeled after those used to evaluate sensitivity to gaze shifts and joint attention in infants and toddlers (Bedford et al., 2012; Falck‐Ytter, Fernell, Hedvall, von Hofsten, & Gillberg, 2012). There are 82 videos in which a single female actor looks straight into the camera as if making eye contact with the participant, executes a gaze shift to one of many possible objects, holds her gaze on the object, and then shifts her gaze back to the camera. Both the Gaze Perception and Gaze Following stimuli have been validated for use in a 4AFC paradigm. These stimuli are available for download and research purposes on Databrary.org (http://doi.org/10.17910/b7.884). Finally, we provide timestamps for 19 40‐second film segments that we extracted from two commercial entertainment movies about adolescents, including *Clueless* and *Ten Things I Hate About You*. These segments feature social interactions that involve many eye gaze shifts and are matched for the number of faces and percentage of time faces are present as well as emotional intensity ratings. Altogether, these stimuli were independently validated by nearly 200 adult raters.

## 4 METHODS

### 4.1 General methods

#### 4.1.1 Human subjects

##### Models

The actors were 16 adults (11 female) who ranged in age from 20–48 years. We did not ask the actors to self‐report their racial or ethnic identity. As a result, we can only provide a general overview of the racial characteristics of the actors who are mostly White; however, several are Black or East‐Asian. Many of them are acquaintances of lab members. The actors all provided consent for their photos to be taken and used for research purposes.

##### Raters

A total of 194 undergraduate students participated in these studies to test the stimuli at various stages of development. Participants gave written informed consent to participate using procedures approved by Pennsylvania State University's (PSU) Institutional Review Board. They were recruited from the PSU Department of Psychology Undergraduate Subject Pool and earned one credit per hour for completing the experimental procedures.

##### Procedures

The procedures were executed on a Dell Latitude E6540 computer with a 15.6‐in. screen using E‐prime 2.0 software. The digital images and videos were recorded using a Fujifilm FinePix S4200 digital camera.

##### Gaze perception stimuli

These stimuli were inspired by a previous study of eye gaze perception (Riby et al., 2013). Each stimulus consists of a color digital photograph of an actor directing his or her gaze at a target object in a complex scene (see Figure 1). There were 26 different scenes including kitchens, offices, living rooms, and bedrooms. The camera distance and lighting were not systematically controlled in these images. The objects in each image were semantically related to the scene (e.g., paper towels, mugs, and bowls in a kitchen scene). Therefore, the objects were not systematically balanced across scenes. In 50% of the original images, actors were instructed to direct both their head and eye gaze to the target object. For example, the actor oriented their head and chin together with their gaze to be in line with the target object (see Figure 1a). In the other 50% of the original images, the actor was instructed to position their head centrally and only direct their gaze to the object so that the head/chin and gaze cues were not spatially aligned (see Figure 1b). In addition to the target object, there was always a *plausible nontarget object*(i.e., spatially close to the target object but not gazed at), and several *implausible objects*(i.e., spatially distant from the target object and not gazed at) in each image.

##### Stimuli

All of the scenes were photographed indoors. Production of these images occurred in two batches. The first batch included 218 photos of 8 actors (5 female) and 19 different indoor scenes (offices, restaurants, houses). The second batch of stimuli consisted of 176 photos of 7 actors (5 female) in 12 different indoor scenes. All the images are standardized for size (1,440 × 1,080 pixels) and resolution (300 dpi).

### 4.2 Procedure

#### 4.2.1 Label generation

To identify the most frequent label for each target object, a group of 12 adults looked at each image on the computer for an unlimited amount of time and were instructed to generate a label for the target gazed‐at object. They did so for each of the 218 images in batch 1. Only images in which the target object was labeled consistently by 50% of participants with the same word were advanced to the next stage of testing. A total of 151 images met this criterion and 67 were excluded at this stage. The rationale for eliminating these items was that either there must not have been sufficient visual information in the gaze cues to consistently identify the target object or the target object was not easily namable. Using this same procedure, an additional 12 adults generated labels for the target objects in each of the 176 images in batch 2. A total of 153 images met the labeling criterion in this batch and 23 images were excluded at this stage. As a result, we had 151 remaining items from batch 1 and 153 items from batch 2 that had ≥50% agreement on the label describing the target gazed‐at object. For the each of these items, the researchers generated three additional labels that corresponded to the plausible nontarget object and two of the implausible nontarget objects to optimize the images for a 4AFC.

#### 4.2.2 Validation of 4AFC task

Responses from two new groups of adults were tested in response to these stimuli to evaluate accuracy in the 4AFC version of the task. A group of 120 adults tested the first batch of 151 stimuli and a separate group of 36 adults tested the second batch of 153 stimuli. Participants were instructed to look at each image and identify the specific object the actor was looking at from a list of four verbal labels presented on a subsequent screen. Each stimulus image was displayed for 3,000 ms and was immediately followed by a response screen that included the four answers and the question, “What object was the person looking at?” The response screen remained until participants executed a keyboard response for the number (1, 2, 3, or 4) that corresponded with their answer. The possible answers included the target object, the plausible nontarget object, and two implausible objects. The order of the object labels was counterbalanced across the four positions. Following the response, there was a 1,000 ms fixation cross before the next trial began. The task was executed in 3 blocks of randomly ordered trials so that participants could take short breaks between blocks. Participants were given three practice trials with feedback prior to beginning the task. Feedback was not provided during the task.

### 4.3 Gaze following stimuli

Our interest in developing dynamic Gaze Following stimuli was inspired by experimental paradigms that have typically been used in eye‐tracking paradigms with young children and infants (see Bedford et al., 2012; Falck‐Ytteret al., 2012; Navab, Gillespie‐Lynch, Johnson, Sigman, & Hutman, 2012; Senju & Csibra, 2008). In these tasks, videos are created of an actor who looks into the camera, as if making eye contact with the participant in an episode of joint attention, and then shifts their visual attention (using head turns with accompanying shifts in gaze and pointing) to one of several objects (2 to 3 objects) in the scene. Because looking at the face is a prerequisite for following the gaze (Senju & Csibra, 2008), researchers often select trials for analysis in which this behavior can be verified via looking time or eye‐tracking assessment (Bedford et al., 2012; Senju & Csibra, 2008;).

Researchers have used these stimuli to evaluate whether infants and children can follow gaze and understand the referential nature of gaze (Bedford et al., 2012; Brooks & Meltzoff, 2005). Previous findings suggest that 9‐month‐old, but not 10‐month old, infants will follow the motion of head turns even when the eyes of an actor are closed, indicating that there is a developmental change in the ability to understand the referential nature of gaze (Brooks & Meltzoff,^2005^). Research also indicates that by 6‐months of age, typically developing infants and those at high risk for developing autism look at the face in these dynamic stimuli prior to the gaze shift with equal likelihood (Bedford et al., 2012; Senju & Csibra, 2008).

Although the stimuli we created were inspired by those used in these early studies, our stimuli are different in two important ways. First, given our interest in using these stimuli to understand potential atypical gaze following behavior in children and adolescents, we specifically choose to minimize head movements of the actor to ensure that success in the task was related to gaze following and an understanding of the utility of eye gaze cues. Second, we increased the number of objects in the visual scene to make the task more developmentally appropriate for use with older children and adolescents. The infant studies employed 2–4 objects, usually in the bottom two (or all four) corners of the screen. In contrast, we included between 8–9 objects along a single plane on a table in our videos.

#### 4.3.1 Stimuli

These stimuli consist of digital color videos depicting a single adult female actor directing her gaze to one of several objects on a table in front of her. The videos were all filmed in the same office with controlled overhead lighting and a fixed camera position. In all videos, the actor sat on a chair behind a table that occluded her body. In this way, there were no social cues from her body to indicate what she was looking at. The objects were small, namable toys (crayons, car), and common objects (battery, tape). The position of the objects was rotated across videos. As in the Gaze Perception stimuli, each video contained a gazed‐at target object, a plausible nontarget object that was spatially close to the target object but not the target of the gaze, and two implausible nontarget objects that were not spatially close to the target object. Each object appeared equally often as a target object, plausible nontarget, and implausible nontarget object.

**FIGURE 2:**
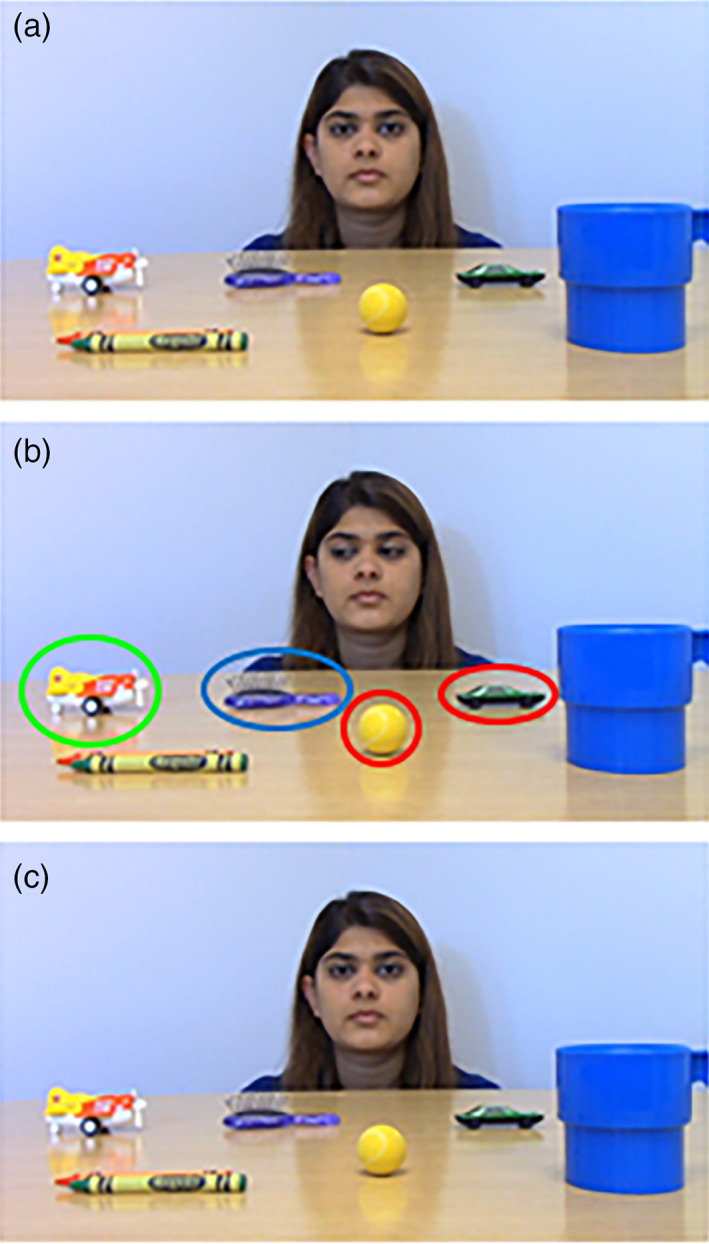
Gaze Following stimuli. The left panel shows still frame images from one of the Gaze Following video stimuli to illustrate the sequence of events. (a) The event begins with the actor looking directly into the camera, (b) she executes a gaze shift to the target object, (c) and shifts gaze back toward the camera. The target object is circled in green, the plausible nontarget object is circled in blue, and the implausible nontarget objects are circled in red

The timing of events in each stimulus was carefully controlled. Each dynamic stimulus begins with the actor looking straight into the camera for 2000 ms as if making eye contact with the participant in an episode of joint attention (see Figure 2a). Next, the actor shifts her gaze toward the target object (~500 ms). The actor then holds her gaze on the target object for 4,000 ms (see Figure 2b). Finally, the actor shifts her gaze back toward the camera (~500 ms) and holds her gaze straight ahead at the camera for another 2000 ms (see Figure 2c). The entire gaze following video lasts approximately 9,000 ms. Importantly, the actor never turns her head during the gaze shifts. As with the Gaze Perception stimuli, the actor gazed at objects positioned to her left, her right, and in the center of the scene in front of her. We created 104 stimuli like this and submitted them to the process of label generation and validation for a 4AFC task.

### 4.4 Procedure

#### 4.4.1 Label generation

We followed the same procedure for identifying labels for the target object for these stimuli as described previously for the Gaze Perception Stimuli above. A group of 17 young adults viewed each stimulus and generated a name for the gazed‐at target object. Participants generated labels for the gazed‐at target object with ≥50% agreement for a total of 90 of the original 104 videos. Researchers generated labels for the plausible nontarget object and two implausible target objects.

#### 4.4.2 Validation of 4AFC task

A new group of 33 adults tested these stimuli in the 4AFC version of the task. Participants were instructed to watch the video and identify the specific object that the actor looked at. Immediately following the video, participants were presented with a response screen that included the 4AFC answers and the question, “What object was the person looking at?” The response screen remained until participants executed a keyboard response for the number (1, 2, 3, or 4) that corresponded with their answer. The possible answers included the target object, the plausible nontarget object, and two implausible objects. The order of the object labels was counterbalanced across the four positions. Following the response, there was a 1,000 ms fixation cross before the next trial began. The task was executed in 3 blocks of randomly ordered trials so that participants could take short breaks.

### 4.5 Movie segments to measure visual social attention

Visual social attention (VSA) is the attentional bias to orient to and look at other people, specifically their face and eyes, and to where they are directing their attention (Birmingham, Bischof, & Kingstone, 2008; Birmingham & Kingstone, 2009). Researchers often study the deployment and modulation of VSA as participants passively view clips from professionally created entertainment movies using eye tracking (see Rice, Moriuchi, Jones, & Klin, 2012). Because of copyright laws, these movie clips cannot be shared. Also, there is very little information about whether and/or how aspects of the movie clips are measured or controlled. Our goal was to identify a set of movie clips that feature social interactions (particularly those between adolescents and adults) that also include many shifts in eye gaze and that are matched on several potentially confounding variables including emotional intensity, number of different faces, and percentage of time faces are on the screen.

#### 4.5.1 Stimuli

These movies segments are all approximately 40 seconds in length (range 40–44 seconds) and were extracted from professionally‐produced entertainment movies from large studios (see Table 1). The movies included *Clueless* and *Ten Things I Hate About You*. In each movie segment, there are between 1–3 faces on the screen at a time. We choose these older movies in the hopes that many individuals who are adolescents now have not seen these movies, to minimize familiarity effects. The starting time for each segment is listed relative to the initial frame introducing the studio (prior to the start of the movie).

**TABLE 1:** Entertainment movie segments for measuring social visual attention

Movie	Scene description	Time on	Time off	Intensity rating
Clueless (1)	Cher and her brother talk on the couch.	0:08:03	0:08:58	2.93
Clueless (2)	Cher, her father, and her brother talk at the dinner table.	0:09:02	0:09:46	3.03
Clueless (3)	Cher's father scolds her in his office.	0:14:39	0:15:19	3.69
Clueless (4)	Cher talks with her brother in the car.	0:16:09	0:16:49	2.25
Clueless (5)	Cher and a friend talk in his car.	0:39:43	0:40:23	2.83
Clueless (6)	Cher and two friends talk in the car.	0:43:40	0:44:24	3.23
Clueless (7)	Cher and her brother tease each other in the house.	0:59:30	1:00:10	2.29
Clueless (8)	Cher and her brother talk on the couch.	1:00:11	1:00:58	2.60
Clueless (9)	Cher and two friends talk in the car.	1:05:01	1:05:45	3.70
Clueless (10)	Cher and a friend talk in the house.	1:15:25	1:16:05	3.59
Clueless (11)	Cher, her brother, and an adult argue at a table.	1:26:49	1:27:33	3.43
Ten Things I Hate (1)	Kat and Bianca argue with their father.	0:13:15	0:13:56	4.40
Ten Things I Hate (2)	Kat and Bianca argue with their father.	0:13:57	0:15:04	3.60
Ten Things I Hate (3)	Bianca and Cameron make plans in the school library.	0:16:04	0:16:45	3.00
Ten Things I Hate (4)	Bianca and Cameron make plans in the school library.	0:16:50	0:17:30	3.20
Ten Things I Hate (5)	Kat and Patrick tease each other in a bookstore.	1:01:33	1:02:14	3.30
Ten Things I Hate (6)	Kat and Patrick talk on a pedal boat.	1:07:50	1:08:31	3.03
Ten Things I Hate (7)	Bianca and Cameron talk to Bianca's father about going to the prom.	1:18:45	1:19:26	3.40
Ten Things I Hate (8)	Kat, Bianca, and Cameron talk on the front porch.	1:27:01	1:27:42	3.00

*Note*: Time on for each segment begins from the initial frame introducing the studio prior to the start of the movie. Emotional intensity ratings were collected on a Likert scale (1 = no emotion, 5 = intense emotion).

#### 4.5.2 Procedure

First the clips were coded for the number and time faces were present on the screen. Faces were coded as “present” during a particular second of the clip if they faced the camera for at least 16 of 30 frames of the second. There were at least 2 raters to code the number of faces in each movie segment. When there were inconsistencies between the ratings, raters met in person to resolve any discrepancies resulting from this process. The total number of seconds any face was on the screen was added up to compute the percentage of total time that faces were on the screen.

To evaluate the emotional intensity of the clips, 37 young adults viewed each movie segment and rated the movie on a Likert scale (1 = no emotion, 5 = intense emotion). Participants were also asked basic comprehension questions about the clips to gauge whether or not they were paying attention.

## 5 RESULTS

### 5.1 Gaze perception stimuli

Because we wanted to identify a set of items from which typically developing adults could reliably perceive the target of directed gaze, we established a minimum accuracy criterion of 75% for each item. Specifically, items that garnered a mean accuracy of less than 75% in the 4AFC task were eliminated from the database. As result, 52 items were rejected from batch 1 and 7 items were rejected from batch 2. There are a combined set of 245 images in the final database. The mean accuracy across all 245 items from the combined 156 adults was 92.8% (*SD* = 6.9%). Data S1 in Supplementary Table S1 includes information for each stimulus item regarding the number of raters, mean accuracy and standard deviation of the rating, sex of the actor, specific actor (e.g., F01, M01), specific scene (e.g., Office_01, Kitchen_03), and nature of the gaze cues (head direction + gaze, gaze only). It also includes the verbal labels for the target object, plausible nontarget object, and two implausible nontarget objects that were used in the 4AFC task. Data S1 in Supplementary Figures S1 –S2 illustrate the mean accuracy ratings for the stimuli plotted as a function of actor (Data S1: Figure S1) and scene (Data S1: Figure S2).

The images are named with a standardized nomenclature that includes the following: the name of the target object, whether the actor is directing eyes alone or head and eyes toward the object, actor specific information (sex and actor number), and a unique number for each image in the database (e.g., 001–245). A subset of these stimuli has been used to test perception and referential understanding of eye gaze cues in young adults who vary in autism‐like traits (Whyte & Scherf, 2018) and in adolescents with autism and age‐, gender‐, IQ‐matched typically developing adolescents (Griffin & Scherf, n.d.).

### 5.2 Gaze following stimuli

As with the Gaze Perception task, we established a minimum accuracy criterion of 75% for each item in the 4AFC task. As result, 8 items were rejected leaving a total of 82 Gaze Following stimuli in the database. The mean accuracy across all 82 items was 92.9% (*SD* = 8.2%). Data S1 in Supplementary Table S2 reports the mean accuracy for each item.

The images are named with a standardized nomenclature that includes the following: the name of the target object, and a unique number for each video in the database (i.e., 001–082). We also include a table with information about the number of raters, mean accuracy rating, and verbal labels for the target object, plausible nontarget object and two implausible nontarget objects that were used in the task for each stimulus item (see Data S1: Supplementary Table S2).

### 5.3 Movie segments

The movie clips are matched for the total number of faces present throughout the scene and on the total percent of time that faces are present in the screen. Data from 7 of the 37 participants were excluded from the analysis because of low compliance with task instructions and poor performance (i.e., more than 1 error) on the comprehension question. The mean emotional intensity ratings for each movie clip are presented in Table 1 along with the start and stop time of each clip and a brief description of the content of the clip.

## 6 DISCUSSION

Here, we describe Eye Gaze FoCuS, a publicly available database of stimuli that are optimized to investigate the perception and referential understanding of shifts in eye gaze. There are three kinds of stimuli. The 245 Gaze Perception stimuli are digital photographs of an adult looking at an object in a complex scene and are designed to test the ability to estimate eye gaze trajectory and interpret the referential intent of the gaze. The Gaze Following stimuli are 82 digital videos of an actor executing a gaze shift to one of many possible objects after engaging in joint attention with the camera/participant and is designed to measure the ability to follow and interpret gaze shifts online. Finally, we provided timestamps for 19 40‐second video clip segments from adolescent‐oriented entertainment movies that are matched in emotional intensity, number of faces and time that faces are present on the screen. These segments involve social interactions with lots of gaze shifts and can be used to measure social visual attention.

The Gaze Perception and Gaze Following stimuli were designed to be used in a 4AFC paradigm to test referential understanding (i.e., psychological intent) of the gaze cue. Each image/video was validated by independent adult raters and only included in the stimulus set if the endorsement of the correct target gazed‐at item was on average ≥ 75%. Researchers can use the stimuli in a passive viewing or the 4AFC paradigm, which will allow them to evaluate referential understanding of the gaze cues and establish a baseline level of performance (25%). Researchers who want to use the identified segments from the entertainment movies to evaluate social visual attention will need to isolate and clip the segments themselves.

Researchers may want to take multiple variables into consideration when selecting subsets of items for their own experiment. For example, in the Gaze Perception stimuli, there is some variation in the accuracy of responses from raters related to the individual actors and scenes. Researchers can use the item‐level information to select specific sets of images that are matched on accuracy across these variables. Also, although the original set of pictures contained an equal number of images with both head and gaze direction information and gaze direction information in isolation, following the validation and down‐select procedures, the final database includes 53.9% images with head direction and gaze cues and 46.1% images with gaze cues only. Depending on the nature of the research question, researchers many need to be mindful about how they select images with regard to this information (see Data S1: Supplementary Table S1). Also, when using the Gaze Following stimuli, researchers may consider that looking at the face of another is a prerequisite of gaze following behavior (see Senju & Csibra, 2008). As a result, many researchers who employ Gaze Following stimuli only analyze trials in which they verify that participants look at the face prior to the gaze shift (e.g., Bedford et al., 2012; Senju & Csibra, 2008). Subsequent work using eye‐tracking technology to assess looking time behavior in response to these Gaze Following stimuli will help determine the effectiveness of eliciting gaze to the face prior to the gaze shift.

There are some limitations to consider regarding these stimuli. A central goal in the design of the Gaze Perception stimuli was that they represent complex, human environments that are ecologically valid. As a result, we did not explicitly control camera distance, lighting, or total number of objects in the display. What is controlled is the fact that a target object, plausible nontarget, and implausible nontarget objects exist in each image. Second, there is only a single actor in the Gaze Following videos. Therefore, impairments in this task could result from difficulty processing actor‐specific gaze cues (difficulty seeing her eyes) or processing gaze cues more generally. Finally, because of copyright laws, we are not able to provide the actual video segments from the entertainment movies. Researchers will have to extract the segments based on the timestamps that we provided. It will be important for researchers to compare segments to verify that they are, indeed, the same.

## 7 CONCLUSION

By making the Eye Gaze FoCuS database available to researchers, this will support the study of visual sociation attention and referential understanding of eye gaze cues and shifts in eye gaze in particular. This database will enable researchers to study the developmental emergence of the perception and interpretation of these critical nonverbal social cues as well as atypical variations in this trajectory. For example, adolescence may be an important time when sensitivity to eye gaze cues is changing. Eye gaze cues are relevant for signaling social status, aspects of theory of mind, and deception, all of which may all take on new meaning in the peer‐oriented relationships of adolescence.

Using the stimuli in the FoCuS database, future researchers can develop a better understanding of phenotypic variations in eye gaze processing in autism, social anxiety, and schizophrenia and determine whether atypical processing of this critical social information is an endophenotype for any of these disorders. The focus on eye gaze cues with explicit strategies for assessing referential understanding of these cues will begin to fill in the gaps in the current literature and allow for nuanced questions about how people perceive and interpret shifts in eye gaze to be addressed. These stimuli could also be used to evaluate the effectiveness of therapeutic interventions that target gaze behaviors and referential understanding of gaze in each of these disorders.

## CONFLICT OF INTEREST

The authors declare no conflicts of interest.

## Supporting information


**Data S1**: Supporting information.
