# Supplementary material for: Measuring sensitivity to eye gaze cues in naturalistic scenes: Presenting the eye gaze FoCuS database
Source: Int J Methods Psychiatr Res. 2020 Jul 14;29(4):e1833. doi: 10.1002/mpr.1833 (PMC7723179; doi:10.1002/mpr.1833)
Supplement: Supplementary file 1 — Data S1: Supporting information. [file MPR-29-e1833-s001.zip › MPR_1833_Eye_Gaze_FoCuS_Supp_Figs (1).docx]

**Supplementary Materials**


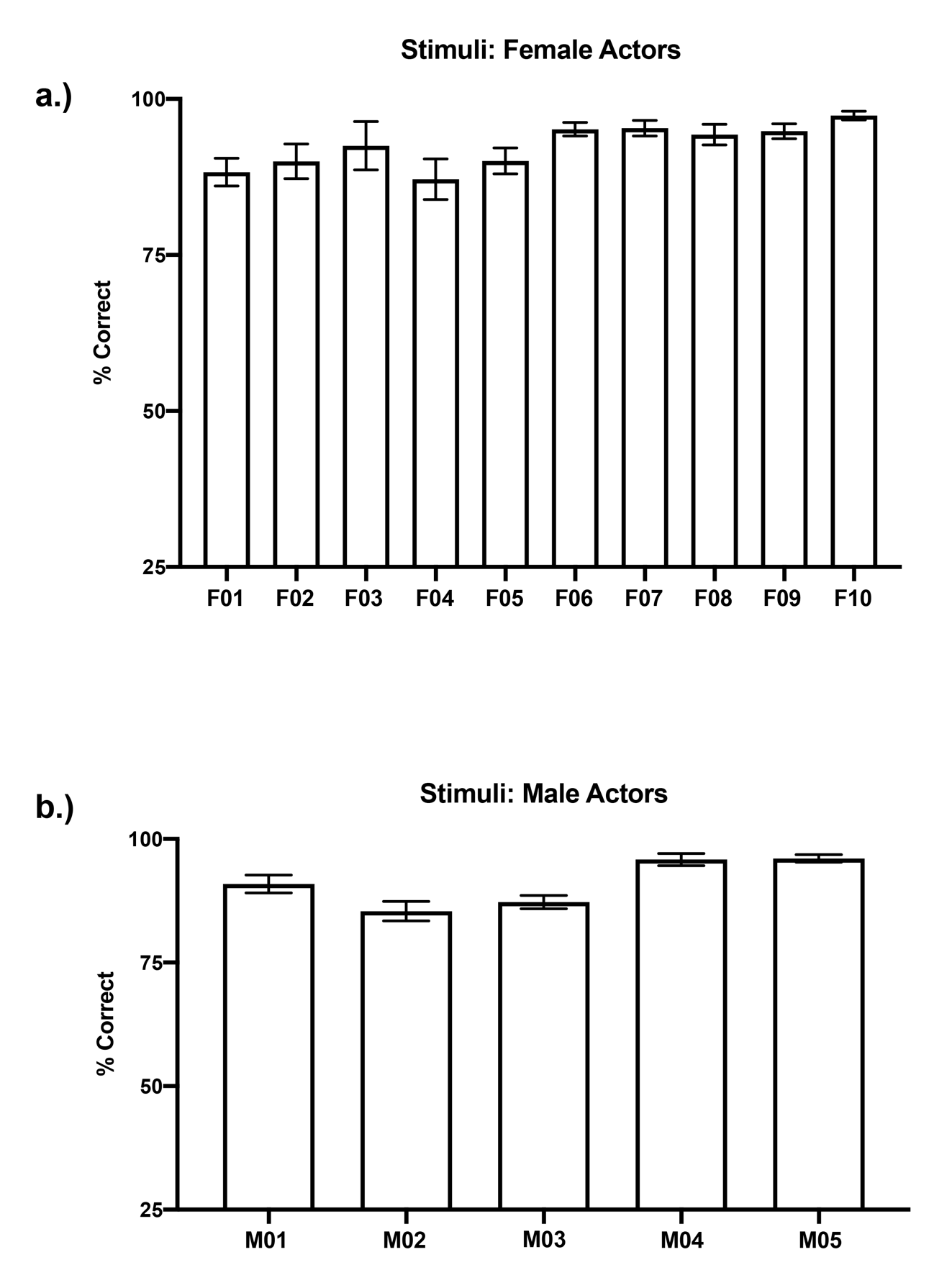


*Supplementary Figure 1.* Mean accuracy (% correct) in Gaze Perception 4-alternative forced choice task to identify gazed-at target items plotted as a function of actor and gender (collapsed across scene). Error bars depict SEM.


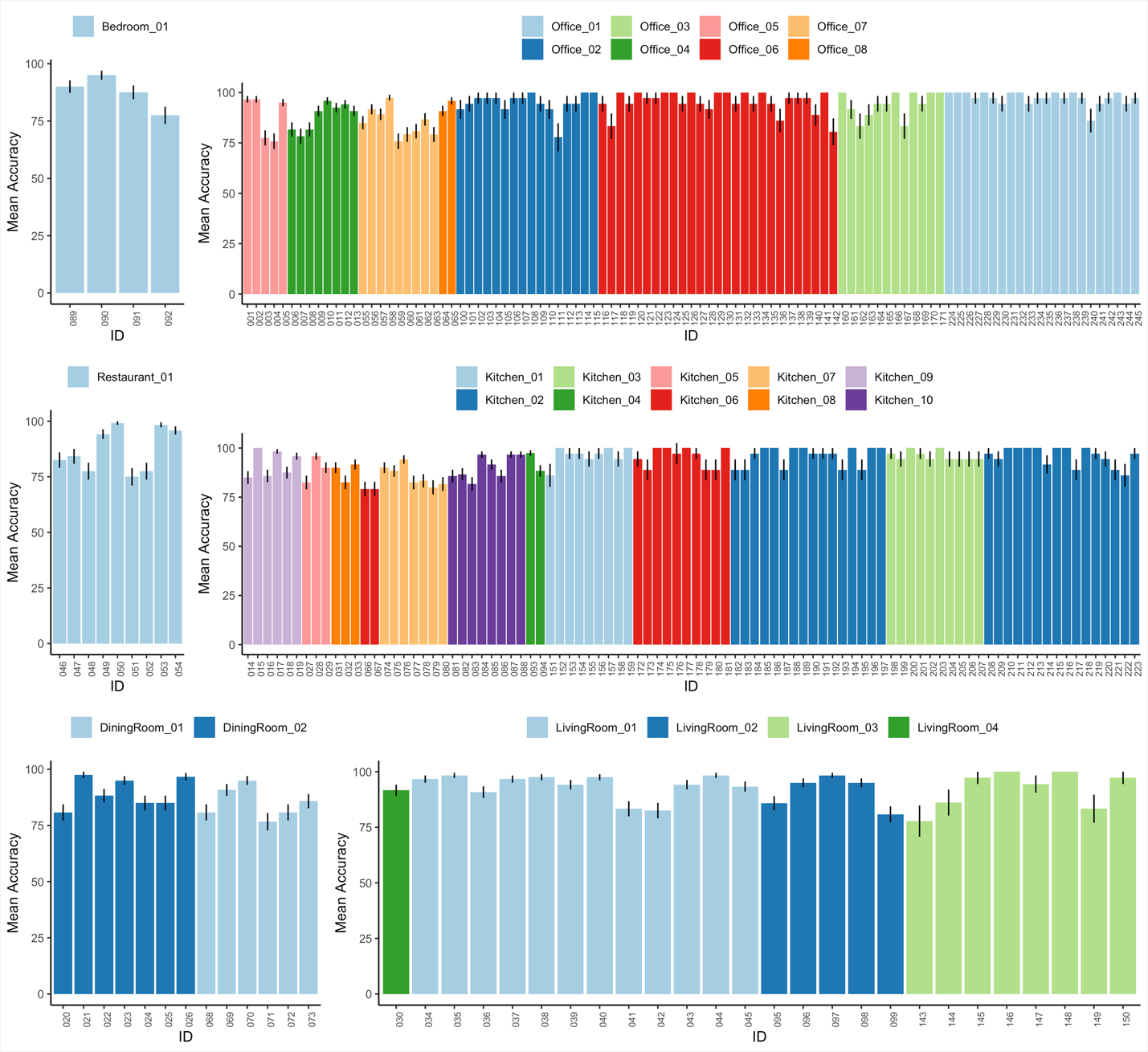


*Supplementary Figure 2*. Mean accuracy (% correct) in Gaze Perception 4-alternative forced choice task to identify gazed-at target items plotted as a function of scene and image. Error bars depict SEM.
